# Cure of Oligometastatic Classic Biphasic Pulmonary Blastoma Using Aggressive Tri-modality Treatment: Case Series and Review of the Literature

**DOI:** 10.7759/cureus.3586

**Published:** 2018-11-13

**Authors:** Jennifer A Lewis, William J Petty, James Urbanic, Eric D Bernstein, Tamjeed Ahmed

**Affiliations:** 1 Internal Medicine, Vanderbilt University, Nashville, USA; 2 Internal Medicine, Wake Forest School of Medicine, Winston-Salem, USA; 3 Radiation Oncology, University of California San Diego Moores Cancer Center, San Diego, USA; 4 Internal Medicine, Providence Cancer Institute, Hood River, USA

**Keywords:** pulmonary blastoma, oligometastatic, radiation therapy, lung cancer, chemotherapy

## Abstract

Pulmonary blastoma is a rare lung cancer classified into three subtypes: classic biphasic pulmonary blastoma (CBPB), well-differentiated fetal adenocarcinoma (WDFA), and pleuropulmonary blastoma (PPB) of childhood. Compared to the other subtypes, CPPB is an aggressive tumor with an overall five-year survival of 16% across all stages. We present two cases of biopsy-proven metastatic CBPB, who have been disease-free for over 10 years since treatment completion. Both patients were treated with surgery to the primary tumor followed by an adjuvant cisplatin-based chemotherapy for four cycles and thoracic radiation. One patient relapsed shortly after the completion of thoracic radiation with brain metastases and underwent craniotomy, gamma knife radiosurgery (GKRS), and whole brain radiation therapy. The other patient presented with synchronous pelvic metastases and underwent metastasectomy after the completion of chemotherapy but before the initiation of thoracic radiation. We review the literature regarding surgical, chemotherapeutic, and radiation treatment for patients with metastatic pulmonary blastoma. Based on our experience and review of the existing case reports, aggressive tri-modality treatment including surgery, chemotherapy with a cisplatin backbone, and a definitive treatment of oligometastatic lesions amenable to local therapy including resection or radiosurgery is reasonable to consider for medically fit patients with CBPB.

## Introduction and background

Pulmonary blastoma is an exceedingly rare type of lung cancer, comprising only 0.25% to 0.5% of all lung malignancies [1-2]. Barrett and Barnard first reported this tumor in 1945 and referred to the malignancy as “embryoma of the lung” due to its histologic resemblance to the first-trimester fetal lung [3-4]. In 1961, Spencer suggested the name “pulmonary blastoma” [5]. He hypothesized that the tumor may originate from a pluripotent stem cell of the pulmonary blastema similar to the Wilms’ tumor from the renal mesenchymal blastema. The histogenesis of this unique tumor has been debated among experts for decades [6-13].

Today, these malignancies are divided into three separate, histologic categories: pleuropulmonary blastoma (PPB), classic biphasic pulmonary blastoma (CBPB), and well-differentiated fetal adenocarcinoma (WDFA) [1]. PPB is a pediatric lung malignancy comprising the mesenchymal tissues and has been found to be associated with trisomy 2. The World Health Organization (WHO) classifies CBPB as a carcinoma with pleomorphic, sarcomatoid, or carcinomatous elements and WDFA as a rare variant of an invasive adenocarcinoma of the lungs [1]. As the name suggests, CBPB has a biphasic histology, consisting of malignant epithelial and stromal cells. The epithelial components are described as being tubular adenocarcinoma with a glycogenated cytoplasm [1]. The stroma is characterized by loose, undifferentiated mesenchymal cells with varying degrees of nuclear atypia. By contrast, WDFA solely comprises malignant epithelial tissues. On a molecular level, WDFA and CBPB are frequently associated with p53 mutations with or without p53 protein overexpression [1].

Approximately 80% of patients with CBPB and WDFA have a history of tobacco use, and most patients present in their fourth decade of life. There is a slight male predominance in CBPB with a 1.5:1 male to female ratio, whereas in WDFA, there is a female predominance of 3:1. Patients with CBPB generally present with symptoms, and tumors average about 10.1 cm in diameter at presentation [1]. WDFA generally presents as small tumors that may be asymptomatic nodules. When present, symptoms are similar to other lung cancers and include a cough, chest pain, hemoptysis, dyspnea, respiratory distress, fever, anorexia, weight loss, fatigue, history of recurrent chest infections, spontaneous pneumothoraces, pleural effusions, and neurological symptoms. Radiographically, WDFA tumors are typically well-defined peripheral lesions on chest radiography and have mixed solid and cystic components on computed tomography (CT) scan [1].

Pulmonary blastomas are an aggressive group of malignancies; two-thirds of patients die within two years, and the five-year survival is 16% [14-15]. Features associated with a poor prognosis include biphasic tumors, p53 gene mutations, tumor recurrence, metastases at initial presentation, and size greater than 5 cm. Few patients with metastatic CBPB achieve long-term survival [16]. We present two cases of metastatic CBPB, who received tri-modality therapy with surgery, chemotherapy, and radiation and are now disease-free for more than 10 years since treatment completion.

Case #1

**Figure 1:**
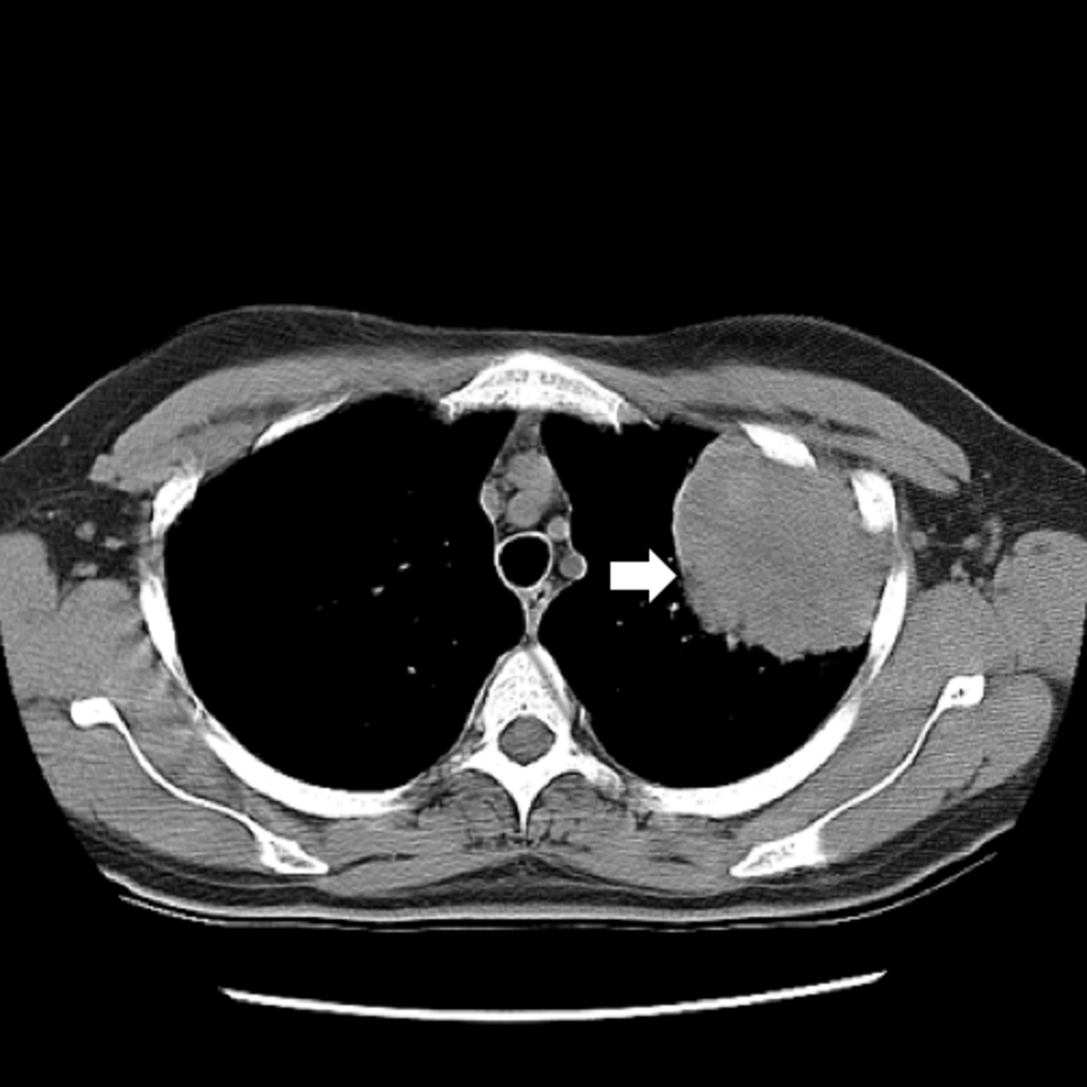
Case 1: chest imaging Computed tomography of the chest at presentation showing a 7 x 5-cm left upper lobe mass.

In 2006, a 38-year-old Caucasian female in excellent health presented with left-sided pleuritic chest pain, cough, intermittent hemoptysis, and seven-pound weight loss. Her medical history was notable for a history of a spontaneous pneumothorax for which she had undergone pleurodesis and bleb resection in 2000 and a 22-pack-year smoking history. A CT scan of the chest demonstrated a 7 x 5-cm left upper lobe lung mass abutting the pleura (Figure 1). Positron emission tomography (PET) demonstrated central necrosis within the lung mass, hypermetabolic activity around the periphery (standardized uptake value; SUV = 14.9), and an increased uptake in the ipsilateral hilar lymph node (SUV = 5.1). Pre-operative brain magnetic resonance imaging (MRI) revealed no evidence of brain metastases. CT-guided biopsy of the mass showed a poorly differentiated carcinoma. 

**Figure 2:**
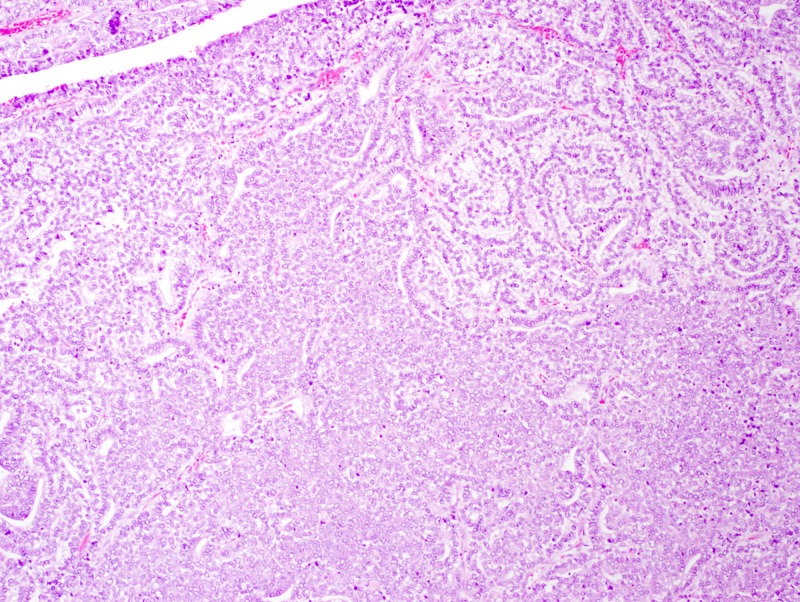
Case 1: lung biopsy Biopsy from the lung mass demonstrated typical features of classic biphasic pulmonary blastoma including mixed epithelial and mesenchymal differentiation with the epithelial component forming glands composed of columnar cells with a clear cytoplasm, while the mesenchymal component showed solid sheets of blastema-like cells

She underwent a left upper lobectomy with chest wall resection and mediastinal lymph node dissection. Surgical pathology revealed a 9.5-cm tumor with clear margins, and four of 11 lymph nodes were positive for metastases. She was pathologic stage IIIA (pT3N2M0). Histological examination demonstrated the typical features of CBPB, including mixed epithelial and mesenchymal differentiation with the epithelial component forming glands composed of columnar cells with clear cytoplasm, while the mesenchymal component showed solid sheets of blastema-like cells (Figure 2). The tumor cells stained for thyroid transcription factor 1 (TTF-1) and low molecular weight cytokeratin and exhibited patchy staining for cytokeratin-7, but not for cytokeratin-20, CD56, or synaptophysin. The patient underwent an adjuvant chemotherapy with cisplatin 50 mg/m^2^ on days 1 and 8 and vinorelbine 25 mg/m^2^ on days 1, 8, 15, and 22 every 28 days. She completed four cycles with frequent dose reductions due to neutropenia and dehydration, and her course was complicated by refractory nausea/vomiting requiring a two-day hospitalization. Following the completion of adjuvant chemotherapy, imaging indicated no recurrence of her disease, and she was referred to radiation oncology for consideration of thoracic radiation. She completed 50.4 Gy in 28 fractions. 

**Figure 3:**
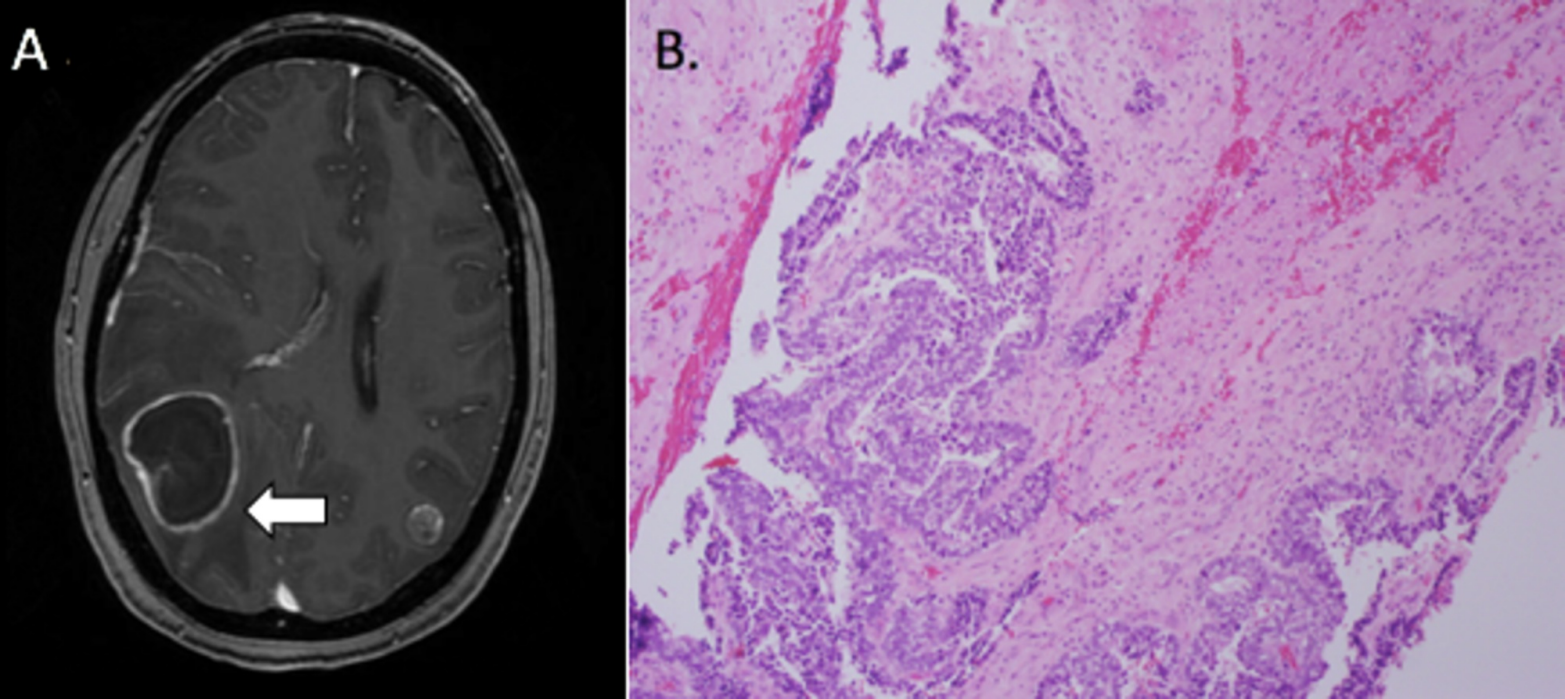
Case 1: brain metastasis (A) Brain magnetic resonance imaging at the time of brain metastasis with right 4 x 3.4 x 3.7-cm temporo-occipital cavitary lesion, left parieto-occipital 1.0 x 1.1 x 1.0-cm lesion, vasogenic edema, and right to left midline shift. (B) Biopsy from the brain metastasis consistent with classic biphasic pulmonary blastoma.

Eight days after the completion of adjuvant radiation therapy, she developed headaches, blurry vision, and dizziness. Magnetic resonance imaging (MRI) of the brain revealed an enhancing right temporo-occipital cavitary mass measuring 4 x 3.4 x 3.7cm, an enhancing left parieto-occipital cavitary mass measuring 1.0 x 1.1 x 1.0cm, vasogenic edema, right to left midline shift, and subfalcine herniation (Figure 3A). She was started on steroids and emergently referred to neurosurgery. She then underwent a right parietal craniotomy for gross total resection of the posterior temporal enhancing mass, followed by gamma knife radiosurgery (GKRS) to the right resection cavity (20 Gy to the 50% isodose line) and left posterior-parietal lesion (20 Gy to the 50% isodose line). Pathology confirmed CBPB (Figure 3B). Additional systemic therapy was initiated with docetaxel, but the patient suffered a severe allergic reaction to the docetaxel and further chemotherapy was declined.

Four months later, she presented with falls and worsening headaches. Brain MRI was consistent with the central nervous system (CNS) recurrence in the right parietal region and a new left frontal lesion. She then underwent a redo right temporoparietal craniotomy followed by GKRS (19 Gy to the 50% isodense line) and whole brain radiation (WBRT; 37.5 Gy in 15 fractions). Pathology again was consistent with metastatic CBPB. Over the next two years, routine surveillance revealed brain lesions concerning for recurrence versus radiation necrosis. She underwent a left frontal craniotomy and left parietal craniotomy. Pathology from the final two craniotomies revealed residual necrosis with no malignancy identified. The patient has had no evidence of disease recurrence for more than 10 years since completing treatment and maintains an excellent quality of life. She continues to be followed with CT imaging of the chest and MRI of the brain on an annual basis.

Case #2

A healthy 29-year-old Caucasian woman with no significant past medical history presented in September 2007 with a persistent cough and dyspnea. Three months prior, she had developed a dry cough during a two-week trip to semi-rural Brazil. Over the next three months, she developed fatigue, decreased appetite, wheezing, and a sensation of dyspnea that “felt like her lung was collapsing.” She was a lifelong non-smoker with minimal secondhand smoke exposure and no history of alcohol abuse or illicit drug use. She lived most of her life in Western Oregon but had spent three months in Southern California. Physical exam was unremarkable other than decreased breath sounds in the left posterior lung fields. She was sent for a chest X-ray that showed a large density in the left chest, which abutted the posterior pleural surface. 

**Figure 4:**
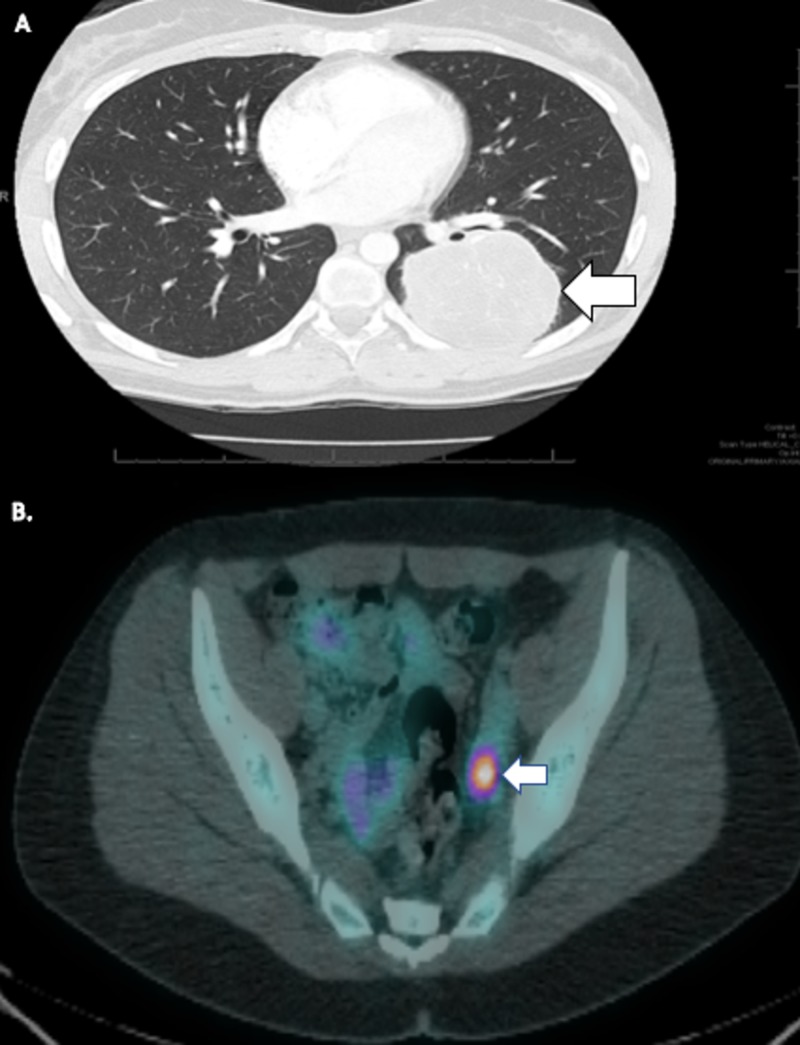
Case 2: baseline imaging (A) Computed tomography of the chest with a 7.7-cm left lower lobe mass. (B) Staging positron emission tomography/computed tomography scan showed a mildly increased uptake in the left adnexal area.

CT scan (Figure 4A) confirmed a large 7.7-cm left lower lobe mass with a sharply demarcated, rounded peripheral margin abutting the chest wall. This partially encased the left lower lobe bronchus and surrounded the left descending pulmonary artery. It was heterogeneous and vascular. Bronchoscopy was performed, but the tissue obtained was non-diagnostic. Staging with whole-body PET/CT scan and MRI of the brain was completed. The left lower lobe lung mass showed extensive fluorodeoxyglucose (FDG) avidity and central necrosis. There were multiple mediastinal lymph nodes with an increased uptake. The left adnexal area had an area of mildly increased uptake but no corresponding mass on CT (Figure 4B). Brain MRI was negative for metastasis. 

Mediastinoscopy was performed, and lymph nodes were negative for malignancy. The patient was taken for thoracotomy and resection of the tumor. Pathology showed a 9-cm tumor with both epithelial glandular and high-grade mesenchymal components. The bronchial surgical margin was positive for neoplasm, and there was involvement of a level 11 lymph node with a poorly differentiated, sarcomatous component. Chromosomal testing for i12p was negative, and serum alpha-fetoprotein (AFP) and beta human chorionic gonadotropin (hCG) levels were normal, effectively ruling out a germ cell tumor. Special staining was negative in both components for mucin, epithelial membrane antigen (EMA), HHF-35, desmin, S-100, chromogranin, and cytokeratin 7 & 20. Vimentin and synaptophysin were both positive in the solid component but negative in the glandular. The keratin AE1/AE3, CAM 5.2, and TTF-1 stains were positive in the glandular component and negative in the solid. Both components stained positive for P63 and mib-1. Pathology was consistent with CBPB, and outside pathology, consultation was in agreement. 

**Figure 5:**
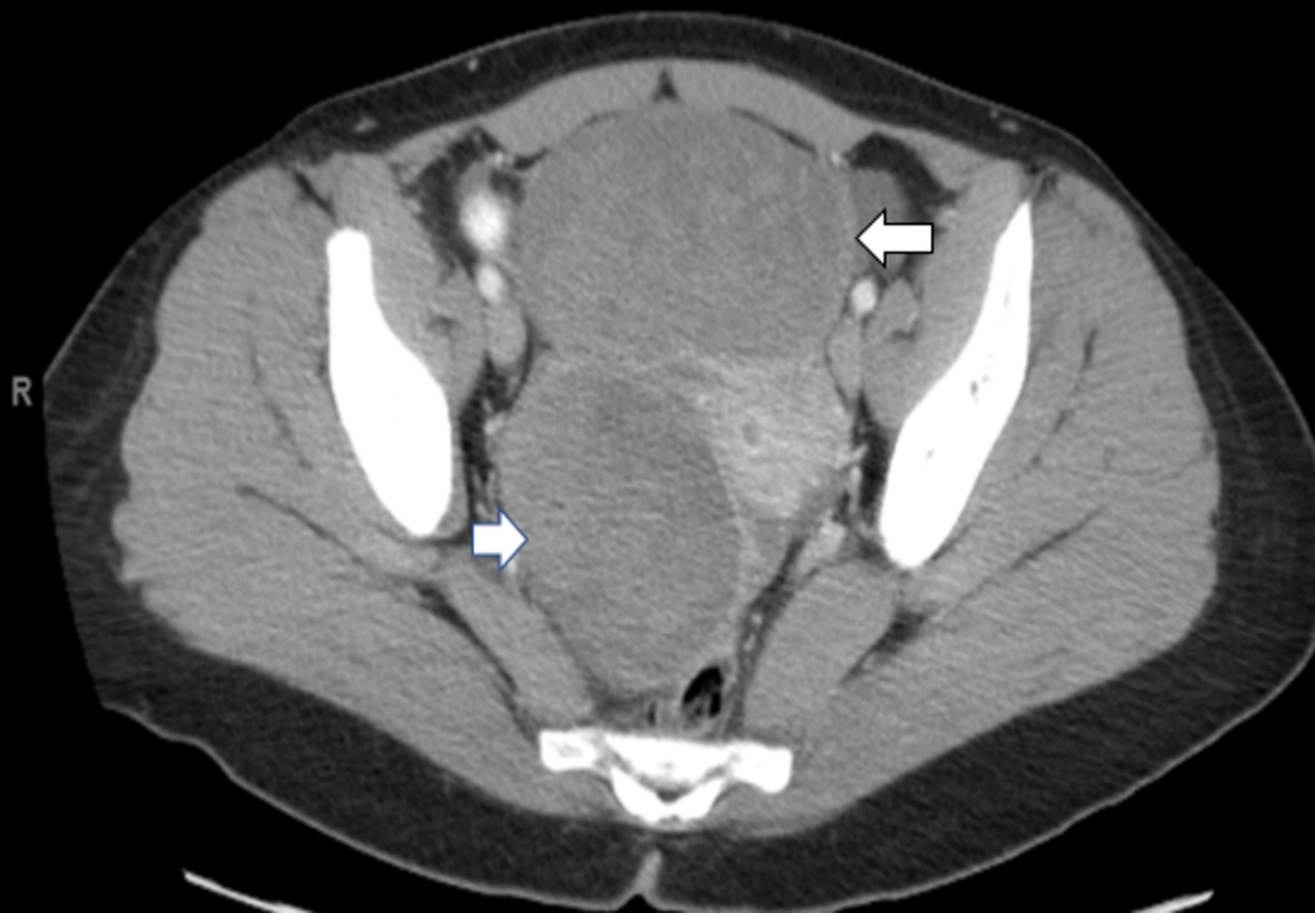
Case 2: imaging of the pelvis Magnetic resonance imaging of the pelvis was obtained six weeks after her initial positron emission tomography/computed tomography to evaluate the areas of hypermetabolic activity. This showed metastases to bilateral ovaries. The anterior mass was 9.2 x 7.9 x 9.1 cm and the posterior mass was 6.3 x 5.6 x 5.6 cm.

MRI of the pelvis was obtained six weeks after her initial PET/CT to evaluate the FDG-avid areas. This showed two new large masses suspicious for metastatic disease to the bilateral ovaries (Krukenberg tumors). The anterior mass was 9.2 x 7.9 x 9.1 cm, and the posterior mass was 6.3 x 5.6 x 5.6 cm. Repeat MRI of the brain and CT of chest/abdomen/pelvis confirmed the large pelvic masses with enlarged periaortic lymph nodes, but there were no other signs of metastatic disease (Figure 5). 

She was admitted to the hospital for chemotherapy consisting of cisplatin 20 mg/m² days 1-5, ifosfamide 1,200 mg/m² days 1-5, and etoposide 75 mg/m² days 1-2 (VIP). She completed a total of four cycles. In March 2008, she underwent laparotomy with resection of the ovarian masses. A necrotic 1 x 2-cm tumor was present on the left ovary, and a smaller irregular area was present on the right. Pathology showed a necrotic nonviable tumor, which resembled the sarcomatous component of a pulmonary blastoma.

Once she recovered from surgery, she was treated with 59.49 Gy of radiation in 33 fractions to the area of the involved level 11 lymph node and a positive bronchial margin. She has been monitored closely with PET/CT and/or CT scan and has had no evidence of recurrence. She became pregnant four months after completion of radiation without any fertility interventions and delivered a healthy child nine months later. She has been doing well since, with no evidence of recurrence over 10 years out from the end of her treatment. 

## Review

Due to the aggressive nature of the disease, there are very few cases in the literature of patients with metastatic pulmonary blastoma who have survived [16-17]. Below we review what is available in the current literature for the treatment of pulmonary blastoma. Interpretation of the existing literature is challenging due to the fact that subtyping of the pulmonary blastoma into the distinct categories of CBPB, WDFA, and PPB did not occur until 1999. We will focus on the treatment for CBPB, similar to our patients, when histology subtype is known. More data are needed to help guide treatment decisions of CBPB, given the extreme rarity and the poor outcomes associated with this malignancy.

Surgery

Surgery has been the standard of care since Barrett and Barnard described the first case of pulmonary blastoma in 1945 [4]. The patient was alive and with no evidence of a disease 15 years after the resection for a localized disease [5]. Surgery has been further supported by numerous case reports and reviews [5-6,18-23]. The two-year survival rate in 1968 was reported to be 55% after surgical resection in a small cohort of nine patients with a clinically localized disease [24]. A more recent case of a patient with locally advanced CBPB (Stage IIIB) underwent surgical resection of the primary tumor after neoadjuvant chemotherapy and was alive at 35 months without any evidence of disease [25]. This compares favorably to a historical control of patients who did not undergo surgical resection and had a median survival of 5.5 months [25].

Surgical resection for metastatic pulmonary blastoma has a long historical context. In 1959, Peacock described the first patient found to have widespread metastatic pulmonary blastoma involving the retroperitoneal and mesenteric lymph nodes, liver, pancreas, kidneys, and adrenal glands on autopsy [18]. One of the first reports of aggressive surgical treatment of pulmonary blastoma is by Kern et al. in 1976 [26]. The authors describe a patient who underwent five thoracotomies with the resection of six tumors within a 19-year period, who was alive and well with no evidence of a disease [26]. Another case reported a 29-year-old female who underwent a left pneumonectomy for the primary tumor [27]. Over the following few years, she underwent several breast and axillary excisions for regional metastases and was reportedly alive and well 16 years later [27].

Surgery may even be beneficial for distant metastases. Kouvaris et al. report a patient who developed a solitary CBPB brain metastasis and underwent craniectomy with partial resection followed by accelerated hypo-fractionated radiotherapy [28]. The patient survived 15 months after partial resection and clinically improved during this time [28]. Another patient who developed renal metastases underwent a laparoscopic nephrectomy after neoadjuvant chemotherapy with sorafenib and was found to have no evidence of disease approximately eight months after resection [29]. These few cases, in addition to the case we present, suggest surgical resection may improve outcomes in patients with an oligometastatic disease. Again, it is important to keep in mind that the interpretation of some of the early cases is limited due to the lack of an identified pulmonary blastoma subtype.

Chemotherapy

There is no consensus or well-established chemotherapy regimen for metastatic pulmonary blastoma. Table 1 provides a comprehensive list of chemotherapies used in the metastatic setting from the published studies reviewed. Single-agent chemotherapy was initially tried with no clinical or objective response. In 1968, Hocholzer was the first to use an adjuvant, actinomycin D, in a 13-month-old female with no objective response, although this tumor was likely a pleuropulmonary blastoma [30]. A review published around the same time describes the first adult with an inoperable metastatic pulmonary blastoma who was treated with nitrogen mustard and cobalt therapy. The patient died at seven months due to tracheal and superior vena cava obstruction [10]. Vila et al. were the first to use an adjuvant combination chemotherapy with chlorambucil and methotrexate in 1973. This patient died within the first month of chemotherapy treatment [31]. The first reported response to chemotherapy in the metastatic setting utilized the cyclophosphamide, lomustine, vincristine, and bleomycin (COMB) regimen, although there are no details provided as to the degree of response [32]. The rationale for this combination chemotherapy described the activity of nitrosoureas and cyclophosphamide in a broad spectrum of tumors, the synergistic activity of lomustine and cyclophosphamide in the Lewis lung carcinoma, and non-overlapping toxicity profiles of vincristine and bleomycin [32]. Over the following decade, clinicians used various cytotoxic regimens with and without radiation, mostly in the adjuvant setting, which demonstrated little response: (1) methotrexate and bleomycin, (2) methotrexate, thiotepa, vilve, 5-fluorouracil followed by adriamycin, (3) cyclophosphamide, vincristine, actinomycin D, (4) intra-pleural 5-fluorouracil, (5) cyclophosphamide, (6) actinomycin D followed by doxorubicin, cyclophosphamide, methotrexate, and procarbazine [9,22-23,27,33-34]. 

**Table 1:** Treatment of pulmonary blastoma in the literature RT: Radiotherapy; CBPB: classic biphasic pulmonary blastoma; SCLC: small cell lung cancer; RUL: right upper lobe

Year	Author	Age	Gender	Location	Size	Histology	Metastasis	Surgery	Chemotherapy/Radiation	Response	Outcome
1969	Stackhouse et al. [10]	32	Female	Right lung	20 x 26 cm	Pulmonary blastoma	Trachea and vena cava (clinical)	Inoperable	Cobalt-60, nitrogen mustard	None	Death
1969	Chitambar [35]	20	Male	Right upper lobe	Large	Pulmonary blastoma	Hilar, nodules near thoracotomy scar	Right pneumonectomy	Radiation to metastatic nodule near thoracotomy scar; methotrexate	Metastasis responded to radiation	Death, one year after surgery
1973	Vila et al. [31]	50	Male	Right lower lobe	14 cm	Pulmonary blastoma	Right pleura, subcutaneous mass on the right posterolateral chest wall	Right lower Lobectomy	Adjuvant chlorambucil, methotrexate, dactinomycin	None	Death, within 1 month
1976	Peacock et al. [18]	23	Male	Left upper lobe	13 cm	Pulmonary blastoma	Heart, left hemidiaphragm, spine, ribs, intercostal muscles	Left pneumonectomy	"Cytotoxic drugs"	Clinical response: improvement in symptoms	Death, 6 months after presentation
1976	Kennedy et al. [27]	27	Male	Left upper lobe	6 cm	Pulmonary blastoma	Left hilum	Left upper lobectomy	Adjuvant cobalt-60 (4200 rads) to left hemithorax, two seven-week courses of methotrexate, thiotepa, vilve, fluorouracil followed by andreamycin	None	Death, 26 months from initial presentation
1976	McCann et al. [23]	40	Male	Right lower lobe	10 cm	Pulmonary blastoma	Pleura, recurrence at right chest wall	Right pneumonectomy, pleurectomy	Adjuvant radiation to right hemithorax and mediastinum, cyclophosphamide, vincristine, actinomycin D	None	Death, 4.5 months post op
1976	Meinecke et al. [9]	80	Male	Right lung	Large	Pulmonary blastoma	Pleura, chest wall, lymph nodes, peritoneum, adrenals, left kidney, liver, vertebral bodies	None	5-Fluorouracil	None	Death, 1 year after initiation of 5-FU
1976	Kern et al. [26]	29	Male	Right middle lobe	8 cm	Pulmonary blastoma	Mediastinum, diaphragm, left lung, retroperitoneum	Right middle lobectomy with multiple metastasectomies	Radiotherapy with chemotherapy after final thoracotomy	Clinical response	Death, nine years after first cyst removal
1977	Fung et al. [33]	19	Female	Right lower lobe	25 x 18 x 9 cm	Pulmonary blastoma	Generalized metastasis including subcutaneous nodules	Right lower lobectomy	Adjuvant radiation with Cytoxan	None	Death, eight months after onset of disease
1978	Roth et al. [34]	66	Male	Right upper lobe	9 x 7x 5 cm	Pulmonary blastoma	Left scapula, left lung	Right pneumonectomy	Adjuvant actinomycin D x four months followed by radiation (5000 rads) to mediastinum, radiation (4,500 rads) to left scapula metastasis, followed by prednisone, adriamycin, cytoxan, methotrexate, and procarbazine for left lung metastases	None	Death, nine months after initial presentation
1980	Jacobsen et al. [15]	59	Female	Right middle lobe, right lower lobe	6 x 5 x 5 cm	Pulmonary blastoma	Hilar lymph nodes, lung, pleura, suprarenal gland	Right pneumonectomy	Chemotherapy	Unknown	Death at one month
1980	Jacobsen et al. [15]	58	Male	Right lung	8 x 4 x 4 cm	Pulmonary blastoma	Chest wall	Right pneumonectomy	Chemotherapy	Possible	Alive at 15 months
1980	Jacobsen et al. [15]	66	Male	Left upper lobe	10 x 8 x 4 cm	Pulmonary blastoma	Hilar lymph nodes, lung, brain, kidney	Craniotomy	Chemotherapy and radiation	Unknown	Death at nine months after craniotomy
1982	Marcus et al. [11]	73	Male	Left upper lobe	8 cm	Pulmonary blastoma	Recurrence	Lingula resection	Adjuvant multiple drug chemotherapy	None	Death, five months after initial resection
1982	Kummet et al. [36]	31	Female	Right upper lobe	8 x 10 cm	Pulmonary blastoma	Recurrence with right hilar mass and bilateral pulmonary nodules	Right upper lobectomy with lymph node dissection	Adjuvant vincristine, adriamycin, cyclophosphamide; cis-platinum, vinblastine, actinomycin D, bleomycin x 3 cycles	Partial response to adjuvant chemotherapy; no response to second chemotherapy regimen	Death, 25 months after intial presentation
1984	Medbery et al. [37]	56	Male	Right upper lobe	3.5 cm	Pulmonary blastoma	Recurrence in the right lung, liver metastasis, left upper lung	Thoracotomy with incisional biopsy of RUL lesion	Adjuvant vincristine, cyclophosphamide, dactinomycin or doxorubicin on alternate cycles	Partial response at six weeks; 14 weeks showed disease progression	Death at 17 weeks after surgery
1989	Dienemann et al. [12]	34	Male	Left upper lobe	6.5 cm	Pulmonary blastoma	Local recurrence, right lung, mediastinal lymph nodes, stomach, brain	Left upper lobectomy	Chemotherapy and irradiation	None	Death, 22 months after surgery
1992	Vassilopoulos et al. [38]	39	Male	Right lung	Large	Pulmonary blastoma	Mediastinum, right pleura	Right chest exploration and tumor removal	Cytoxan, vincristine , doxorubicin, dacarbazine x4 cycles	Clinical response; no radiologic change in tumor size	Death, five months after initiation of chemotherapy
1994	Suzuki et al. [16]	59	Female	Left upper lobe	Unknown	Pulmonary blastoma	Lung, lymph nodes, chest wall, skin	Left upper lobectomy, thoracotomy x2, and chest wall resection	>30 cycles of cisplatinum, cyclophosphamide, vincristine, vindesine, etoposide, doxorubicin, intrapulmonary mitomycin-C as well as adjuvant chemotherapy, radiation	Partial response, possible complete response	Alive nine years after initial operation
1995	Hasturk et al. [39]	57	Male	Left upper lobe	11 x 12 cm	Pulmonary blastoma	Mediastinum	Inoperable	Cisplatin, etoposide, adriamycin x2 cycles followed by radiation x2 cycles (6,000 rads) followed by two additional cycles of chemotherapy	75% regression	Death, 10 months
1998	Cutler et al. [40]	54	Male	Left upper lobe	17 x 21 x 6 cm	CBPB	Left pleura, pericardium, mediastinal lymph nodes	Left pneumonectomy	Adjuvant radiation in 30 fractions of 6,000 cGy over 8 weeks followed by cisplatin/etoposide x3 cycles	Possibly complete response	Alive at seven years
2002	Zaidi et al. [25]	23	Male	Unknown	8 cm	CBPB	Hepatic	Inoperable	Vincristine, dactinomycin, cyclophosphamide, cisplatinum, doxorubicin	Unknown	Death, at eight months
2002	Zaidi et al. [25]	24	Female	Left upper lobe	15 cm	CBPB	Pleural effusion	Left upper lobectomy	Neoadjuvant vincristine, dactinomycin, ifosfamide, doxorubicin, etoposide, carboplatin	Complete response	Alive at 35 months with no evidence of disease
2002	Surmont et al. [41]	49	Male	Left lung	Large	Initially SCLC, later PB	Developed recurrence, right lung	Inoperable	Cyclophosphamide, doxorubicin, and etoposide; started palliative radiation 20 Gy in five fractions of 4 Gy followed by cisplatin and etoposide; radiation repeated 20 Gy with five fractions of 4 Gy	Partial response	Alive one year after diagnosis
2005	Walker et al. [42]	21	Female	Right lung	Large	CBPB	Lymph nodes, diaphragm, right pleura, bone	Thoracotomy with decortication	Chemotherapy	Unknown	Death, six months after presentation
2006	Liman et al. [43]	27	Female	Right upper lobe	6 cm	CBPB	Lymph nodes, bone, liver, brain	Right upper lobectomy	Vincristine and cyclophosphamide followed by ifosfamide and etoposide	No response	Death, at 17 months
2006	Liman et al. [43]	54	Male	Right lower lobe	6.5 cm	CBPB	Lymph nodes, bone	Right lower lobectomy	Vincristine and cyclophosphamide	No response	Death, at 10 months
2010	Zagar et al. [44]	24	Male	Right upper lobe	7.7 cm	Pulmonary blastoma	Recurrence	Right upper lobectomy, right pneumonectomy	Neoadjuvant radiation (60 Gy) followed by concurrent chemo-RT with cisplatin and etoposide (50 Gy total) in 2 Gy daily fractions; followed by adjuvant cisplatin and etoposide x 2 cycles	Partial response	Alive post op
2011	Van Loo et al. [1]	77	Male	Right upper lobe	6 cm	CBPB	Local recurrence, liver, bone	Right upper lobectomy with lymph node dissection	Sorafenib	Unknown	Died one week after started chemotherapy
2011	Lindet et al. [45]	22	Female	Right lobe	12 cm	CBPB	Liver	Right pneumonectomy, pericardiectomy	Ifosfamide, doxorubicin x6 cycles then doxorubicin x2 cycles followed by stereotactic radiotherapy (40 Gy); carboplatin, vincristine,cyclophosphamide; actinomycin-D, docetaxel; gemcitabine	"Major tumor shrinkage"	Death, 18 months after presentation

In 1982, Kummet et al. presented a chemotherapeutic regimen that achieved a partial response in an adult patient with a metastatic pulmonary blastoma [36]. The patient was a 31-year-old female who presented with a cough and was found to have a large right upper lobe mass. She underwent a right upper lobectomy but was found to have bilateral lung metastases within a year after surgery. She was given vincristine 2 mg, doxorubicin 50 mg/m2, and cyclophosphamide 500 mg/m2 every three weeks due to their effectiveness in sarcomas. She was found to have a greater than 50% reduction in tumor size at three months. She eventually developed local recurrence and died seven months from the initiation of chemotherapy. The authors conclude that patients with large primary tumors (> 5 cm) may benefit from a combination of surgery and chemotherapy [36].

Similarly, Medbery et al. describe a patient with a metastatic disease who achieved an objective response at six weeks with 21-day cycles of vincristine 1.4 mg/m2, cyclophosphamide 45 mg/kg, and dactinomycin 2 mg/m2 or doxorubicin 60 mg/m2 alternating every other cycle [37]. These agents were chosen for their known activity in Wilms’ tumors and rhabdomyosarcoma, which share some histologic resemblance to pulmonary blastoma. However, at 14 weeks after initiation of chemotherapy, the tumor began to increase in size, and the patient died at 17 weeks after starting cytotoxic therapy. The authors state that the four-drug chemotherapeutic regimen may provide a median survival of six months [37].

Other cytotoxic regimens that have achieved some clinical or partial response include: (1) cyclophosphamide 800 mg IV, vincristine 2 mg IV, doxorubicin 80 mg IV, and dacarbazine 400 mg IV on days one and three, (2) etoposide 100 mg/m2 daily for five days and cisplatin 20 mg/m2, (3) cisplatin, etoposide, and adriamycin followed by two cycles of radiation, (4) sorafenib, (5) cisplatin, cyclophosphamide, vindesine, vincristine, etoposide, doxorubicin, and intrapulmonary mitomycin-C, (6) cisplatin and etoposide in addition to radiation for a locally advanced disease, and (7) ifosphamide and doxorubicin [16,29,38-40,45-46].

Neoadjuvant chemotherapy has also shown some remarkable responses. In a retrospective cohort study, two patients with a pulmonary blastoma were treated with a neoadjuvant cisplatin or carboplatin and ifosfamide and were able to undergo surgical resection of the down-staged tumors [25]. A 24-year-old male with an inoperable, recurrent disease was treated with neoadjuvant radiation, cisplatin, and etoposide and had a dramatic response. Following two cycles, the patient was able to undergo a right pneumonectomy [44].

These cases suggest a survival benefit for chemotherapy with or without radiation in the metastatic setting. Koss et al. examined 52 cases of WDFA and CBPB at a single institution in a retrospective cohort and found a mean survival of 11 months in those patients who demonstrated disease recurrence and were treated with chemotherapy and radiation (regimens and doses used are not described) compared to two months in those patients who did not receive any treatment [47]. Due to the rarity of this disease and few cases, it is difficult to estimate an accurate survival in patients with metastatic pulmonary blastoma who are treated with chemotherapy, but the literature suggests a survival advantage with a combination chemotherapy.

Radiation therapy

The role of radiation therapy is not well defined but may prove to be more promising than originally demonstrated. Radiation has been used alone and in combination with chemotherapy in patients with inoperable disease or in an adjuvant setting for those considered high risk for recurrence. The first use of radiation therapy was by Parker et al. in 1965 [19]. The patient was a 33-year-old female who was found to have a 5 x 4 x 4-cm left lung mass with a chest wall invasion. She underwent neoadjuvant radiation therapy with an initial decrease in size but later developed progression. The patient then underwent a left pneumonectomy with a chest wall resection. Two years after her surgery, she was found to have nodules in her right lung that disappeared with radiation. Eight years later, she was found to have a right lower lobe mass for which she underwent surgery. She survived a total of 11 years after her initial resection but eventually died from complications of metastatic disease [19]. Cox et al. employed an adjuvant radiation therapy, which the patient tolerated well and continued to have no evidence of disease [20].

Radiation therapy has demonstrated mixed responses in the metastatic setting. In the late 1960s, Chitambar et al. reported a metastatic nodule noticed eight months post-operatively that responded well to radiation [35]. Another patient survived approximately two years after a repeat thoracotomy in addition to postoperative radiation and chemotherapy. This patient survived for nine years after the discovery of the lung mass with an aggressive tri-modality therapy [26]. However, others have found a little benefit of radiation in combination with chemotherapy for metastatic disease [8,10,22-23,27]. One patient died 4.5 months postoperatively despite adjuvant radiation in addition to radiation and chemotherapy for a recurrent disease [23]. Similarly, a patient who received adjuvant radiation and cyclophosphamide died six months post-operatively [33]. In the early 1990s, authors from a single medical institution reported over 50 cases of pulmonary blastoma, including 13 patients who were either diagnosed with a metastatic disease or later developed recurrent CBPB. They noted that chemotherapy and radiation did not result in long-term survival but did seem to prolong survival [47].

Despite historically limited success, radiation with or without chemotherapy has been employed for metastatic disease in recent years and continues to demonstrate variable responses. There is a recent report of long-term survival with the use of surgery, chemotherapy, and radiation for metastatic disease [16]. Cutler et al. reported a patient with a 9 x 10 x 8-cm tumor in the left upper lobe that extended to the left pleural cavity, pericardium, and mediastinum who was treated with adjuvant chemotherapy and radiation following surgery. This patient was alive seven years after his initial surgery and free of disease [40]. Bosch-Berrera and colleagues describe a patient who was initially unresectable and was treated with cisplatin plus etoposide followed by concurrent chemoradiation (50.4 Gy) with weekly cisplatin. This resulted in a significant downsizing of the tumor, and the patient was able to go for a pneumonectomy [48]. Perhaps the most impressive case utilizing radiation alone was a male who was treated with palliative radiation (20 Gy/5 fractions) for a large, locally advanced pulmonary blastoma. The patient was expected to live only a few weeks, but instead, followed up a month later and was asymptomatic with 75% tumor regression [41]. Similar responses have been observed with the use of chemotherapy and radiation [39,44]. Others in more recent years have had less success with radiation for metastatic disease [25,28,42-43]. Over the course of many decades, radiation has been used in various settings with or without chemotherapy with variable responses.

## Conclusions

Pulmonary blastoma is an exceedingly rare but aggressive malignancy. Surgery is the cornerstone of treatment, but the most efficacious role for chemotherapy and radiation remains unclear due to the rarity of this disease. Each of these patients had multiple poor prognostic features (biphasic histology, size greater than 5 cm, and recurrence) and was treated with surgery to the primary tumor. Both patients received an adjuvant cisplatin-based chemotherapy for four cycles followed by thoracic radiation. Both patients have had an outstanding outcome, and the observations from each case may be valuable, given the extreme rarity of this disease.

Based on our experience and review of the literature, surgery or radiosurgery should be considered in patients with a localized disease and may also be helpful in patients with oligometastatic disease. Systemic chemotherapy with a cisplatin backbone is reasonable to consider in the adjuvant or metastatic setting, while using other agents in combination, such as vinorelbine, ifosfamide, etoposide, and/or doxorubicin may also be efficacious. Radiation, with or without concurrent chemotherapy, has been used in the adjuvant and metastatic settings and should be considered in patients with an oligometastatic disease amenable to radiotherapy. 

A cancer registry of adults with pulmonary blastoma (CBPB and WDFA) is needed to share treatment approaches and patient outcomes as we search for the most efficacious treatments for this rare malignancy. In our limited experience, an aggressive tri-modality treatment including surgery, chemotherapy, and radiation is a reasonable approach in a patient with pulmonary blastoma with an oligometastatic disease amenable to a local therapy as evidenced by these two patients who continue to have no evidence of disease recurrence 10 years after treatment completion.
